# On the stability of microwave-fabricated SERS substrates – chemical and morphological considerations

**DOI:** 10.3762/bjnano.12.44

**Published:** 2021-06-11

**Authors:** Limin Wang, Aisha Adebola Womiloju, Christiane Höppener, Ulrich Sigmar Schubert, Stephanie Hoeppener

**Affiliations:** 1 Laboratory of Organic and Macromolecular Chemistry (IOMC), Friedrich Schiller University Jena, Humboldtstr. 10, 07743 Jena, Germany; 2 Jena Center for Soft Matter (JCSM), Friedrich Schiller University Jena, Philosophenweg 7, 07743 Jena, Germany; 3 Leibniz-Institut of Photonic Technology e.V. (IPHT), Albert-Einstein-Straße 9, 07745 Jena, Germany; 4 Institute of Physical Chemistry, Friedrich Schiller University Jena, Lessingstr. 10, 07743 Jena, Germany

**Keywords:** chemical stability, microwave synthesis, scanning electron microscopy, silver nanoparticles, surface-enhanced Raman spectroscopy

## Abstract

The stability of surface-enhanced Raman spectroscopy (SERS) substrates in different organic solvents and different buffer solutions was investigated. SERS substrates were fabricated by a microwave-assisted synthesis approach and the morphological as well as chemical changes of the SERS substrates were studied. It was demonstrated that the SERS substrates treated with methanol, ethanol, or *N*,*N*-dimethylformamide (DMF) were comparable and showed overall good stability and did not show severe morphological changes or a strong decrease in their Raman activity. Toluene treatment resulted in a strong decrease in the Raman activity whereas dimethyl sulfoxide (DMSO) treatment completely preserved or even slightly improved the Raman enhancement capabilities. SERS substrates immersed into phosphate-buffered saline (PBS) solutions were observed to be rather instable in low and neutral pH buffer solutions. Other buffer systems showed less severe influences on the SERS activity of the substrates and a carbonate buffer at pH 10 was found to even improve SERS performance. This study represents a guideline on the stability of microwave-fabricated SERS substrates or other SERS substrates consisting of non-stabilized silver nanoparticles for the application of different organic solvents and buffer solutions.

## Introduction

Surface-enhanced Raman spectroscopy (SERS) has been developed into a standard analytical tool in chemical and bioanalytical research [1–2]. Its application in chemistry, biosensing, materials science, engineering, and medical diagnostics was successfully introduced and can provide valuable information [1–4]. Since it was discovered in 1974 by Fleischmann et al. that the intensity of Raman signals can be significantly improved when the measurements are performed on roughened silver electrodes [5], an understanding of the SERS enhancement process has been gained from a large number of investigations. This has rationalized the requirements for a good performance of SERS substrates. Van Duyne and others elaborated the fundamental concept of the enhancement process, which was found to be mainly based on the amplification of the electric field component when the illuminating laser irradiation interacts with metal nanoparticles [6]. Suitable nanoparticles consist preferentially of gold, silver, or copper. Additionally, the shape, density, and form of the nanoparticles on the SERS substrates were found to be critical parameters for field enhancement [4,7] and the formation of the so-called hot spots [4]. These hot spots can be created, for example, by nanoparticles forming small gaps which can particularly enhance the Raman intensities. Consequently, a lot of research and development efforts have been devoted to the fabrication of high-performance substrates following a wide range of different design concepts. These include, for example, the assembly of colloidal nanoparticles on the substrates [8], the synthesis of complex nanoparticle structures with tunable interparticle gap sizes [9], the utilization of micro- and nanofabricated structures obtained by lithography techniques [10–12] comprising nanodisk arrays [13], nanoholes [14–15], nanocups [16–17], nanogratings [18], or the customization of readily available structures (i.e., HR-DVD templates) [19]. The aim of these developments is not only the optimization of the nanoparticle arrangement to obtain high enhancement factors, but also to guarantee uniformity, homogeneity, the ease of fabrication, low fabrication costs, as well as the stability of the SERS substrates to enable their universal applicability [7].

The microwave-assisted synthesis of nanoparticles is by now an established method for the synthesis of uniform metal nanoparticles in solution, which can be applied to fabricate SERS substrates by drop-coating and self-assembly procedures [20–22]. Alternatively, monolayers of nanoparticles can be formed directly on substrates by a microwave-assisted one-step fabrication process [23–24] on glass and silicon-based substrates with different shapes (e.g., on flat substrates as well as inside glass capillaries or on scanning force microscopy tips) in a very economic and fast (less than five minutes) coating process. The formed substrates, which are coated with a monolayer of silver nanoparticles, have been demonstrated to be highly reproducible and to perform very well in the detection of analytes, as demonstrated by the detection of tetrahydrocannabinol (THC) down to a concentration of 0.25 nM by utilizing microwave-coated glass capillaries [25]. While this approach relies on ethanol as reducing agent and applies no protecting agents, other protocols employ suitable surfactants and additives to fabricate uniform Ag nanoparticles. These include basic aminoacids (such as ʟ-lysine or ʟ-arginine) and soluble starch or other additives, which act as reducing and protecting agents, respectively [22].

**Figure 1:**
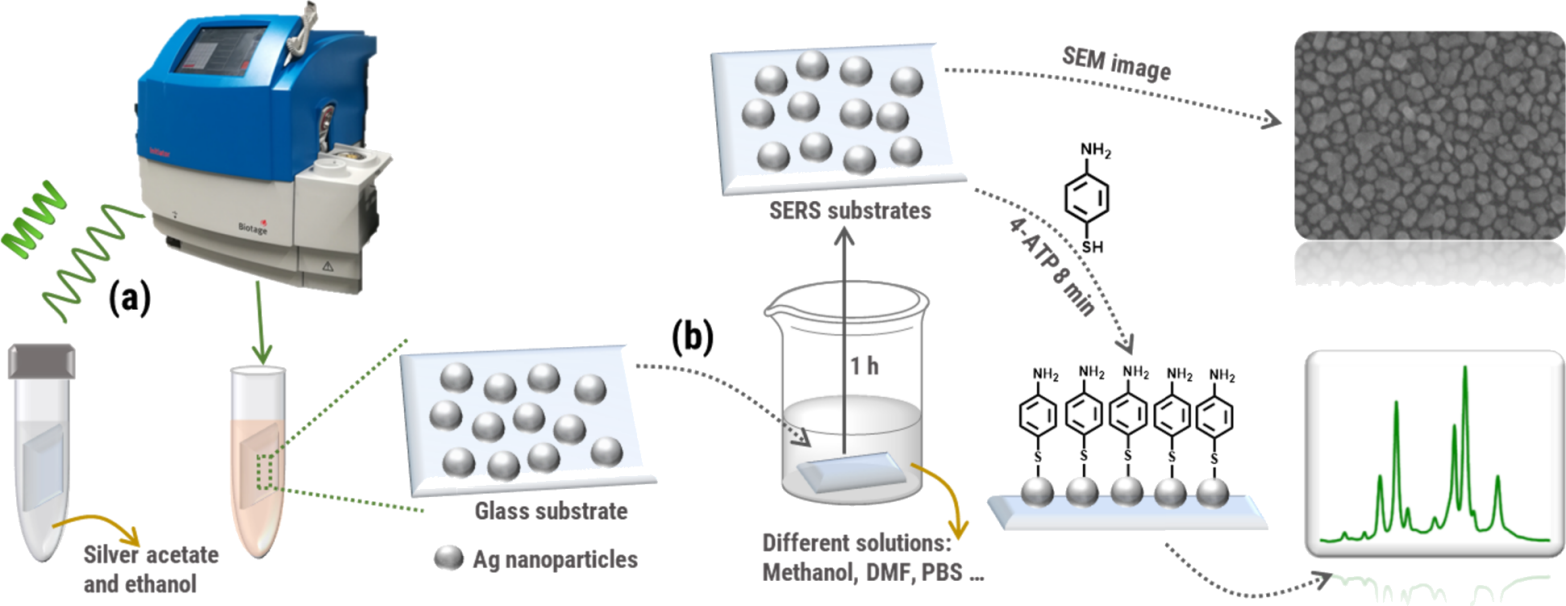
Schematic illustration of the microwave-assisted fabrication of SERS substrates (a) and the experimental pathway for investigating the stability of the SERS substrates in different organic solvents as well as in biological buffer solutions (b).

In our approach, the monolayer of silver nanoparticles is formed by the reduction of silver salt to metallic silver by microwave irradiation in the presence of ethanol utilized as reducing agent (Figure 1a). In practice, the functionalization of the substrates requires only the placement of the cleaned glass supports into a microwave vial containing an aqueous silver acetate precursor co-mixed with ethanol. Subsequently, the closed microwave vials are subjected to microwave irradiation for two minutes.

In the first phase of the microwave irradiation the temperature of the precursor mixture rapidly increases (see Supporting Information File 1, Figure S1 for the temperature diagram of a typical heating process) which initiates the reduction of the silver salt by ethanol. This triggers a short burst of silver nanoparticle nucleation, which forms reaction seeds on the glass substrate. The strong affinity of the silver ions for the free hydroxyl groups of glass surfaces allows for the attachment of the nanoparticles to the substrate and the glass surface of the microwave reaction vial. An accompanied rapid growth phase, resulting from the high microwave absorption capabilities of the seeds, stimulates the further reduction of the available silver ions on the seed surface. For the formation of SERS substrates, the best reaction conditions were determined to result in the reproducible formation of well-performing SERS substrates in a batch process. The main advantages of this microwave-assisted fabrication of SERS substrates are the extremely fast processing time (<5 minutes in total) and the utilization of non-toxic reaction agents which additionally ensure that the obtained SERS substrates are free of impurities and additives that could result in undesired background signals during the SERS measurements. These SERS substrates have been used for SERS investigations and were employed in a large number of analytical measurements in our laboratories since we established the microwave-assisted fabrication process. One critical issue is the stability of the formed SERS substrates and, up to now, only results obtained by the application of the analyte in the form of aqueous or ethanolic solutions were reported. Since specialized applications also involve the deposition of analytes from other organic solvents or, in the case of the investigation of biological samples, even from buffer solutions, a more careful investigation of the performance and stability of the microwave-fabricated SERS substrates is mandatory. For this reason, we investigated this issue and focused on the application of organic solvents and buffer solutions which are frequently used in organic and bio(analytical) applications. Their influence on possible morphological and/or chemical changes of the SERS substrates is studied via scanning electron microscopy (SEM) and their impact on the SERS enhancement capabilities of the substrates is evaluated by Raman spectroscopy (Figure 1b). For this purpose, all treated substrates are carefully rinsed after immersion into the solvents and buffers and are subsequently coated with a self-assembled monolayer of 4-aminothiophenol (4-ATP), which serves as a defined molecular probe layer to determine the SERS activity of the substrates.

## Results and Discussion

**Figure 2:**
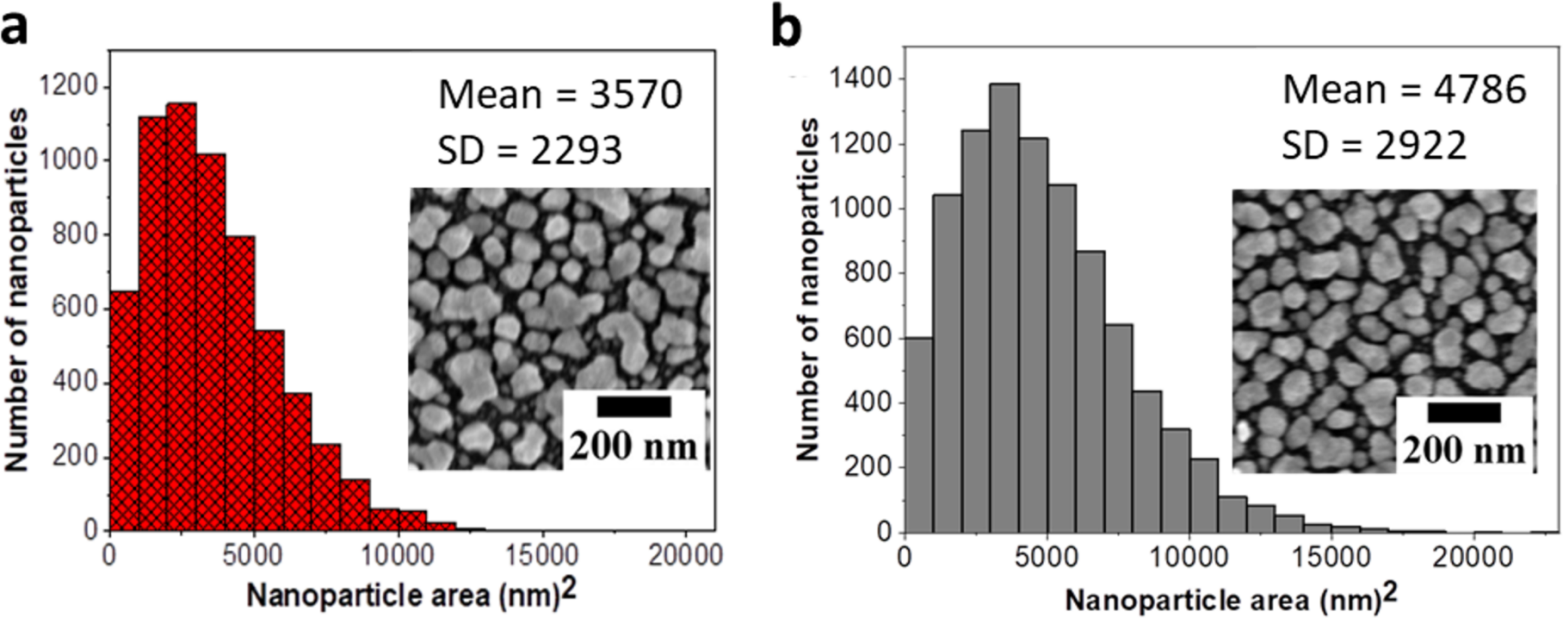
Particle size distributions and SEM images of the microwave-fabricated SERS substrates (a) after synthesis and (b) after immersion in water for 1 h. Data were acquired on different substrates prepared in different batch synthesis processes (*n* = 5). The nanoparticle area represents the projected area occupied by an averaged nanoparticle.

For the performed stability tests, SERS substrates were fabricated by microwave-assisted synthesis in a batch process. All substrates were characterized before the stability tests and revealed the formation of a reproducible monolayer of nanoparticles on the substrate, which is in line with the previously reported procedure. Typically, the Ag nanoparticles synthesized by the microwave-assisted synthesis approach are irregular in shape and well separated (Figure 2a), which supports the formation of hot spots in these nanoparticle films. At the same time, the nanoparticles are rather polydisperse, which is seen as a beneficial feature of SERS substrates as it contributes to the electromagnetic enhancement capabilities of the SERS substrates due to size and nanolensing effects [26–27]. However, this complicates the quantitative analysis of the nanoparticle sizes. In this study, we analyzed the averaged particle size distribution by evaluating the projected area of the particles to determine the particle coverage, as well as the average projected spherical analogue particle size. Even though it has to be considered here that the standard deviations of the determined particle sizes and projected areas are rather high, these values can serve as a first estimation of the particle sizes in the SERS films before and after treatment. Therefore, it can be used in an initial evaluation of the morphological changes observed on the substrates after their immersion into different solvents and buffer solutions in comparison to the respective reference samples.

The projected particle size analysis of the microwave-fabricated SERS substrates (Figure 2a) indicates a reasonable reproducibility of the fabrication process (Supporting Information File 1, Figure S2) and can be used for the evaluation of the morphological changes, as exemplified by the samples which were treated for 1 h with water (Figure 2b). Both samples served as references for the subsequent studies.

**Figure 3:**
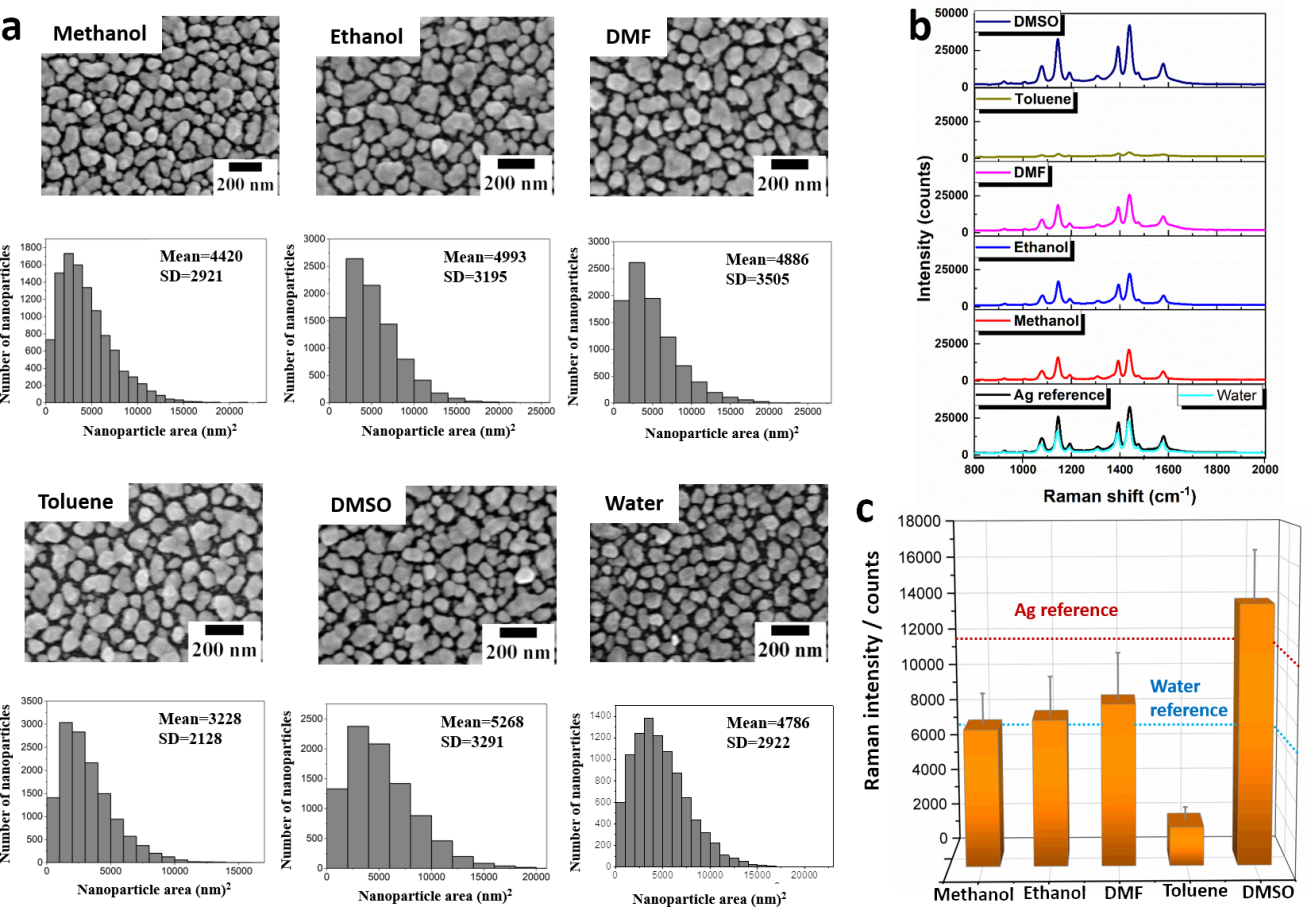
Stability tests of microwave-fabricated SERS substrates for different organic solvents. (a) Morphology of the SERS substrates and corresponding particle size distributions of the SERS substrates after 1 h of immersion into methanol, ethanol, DMF, toluene, and DMSO. The projected areas of the nanoparticles were determined by automated analysis and are summarized in the histograms. Each batch synthesis consisted of three substrates. A total of six substrates from two batches were examined by randomly selecting eight different areas measured on each substrate to evaluate the reproducibility from batch to batch. (b) SERS spectra obtained on the individual SERS substrates of a monolayer of 4-ATP self-assembled on the treated substrates. (c) Error analysis of the 1078 cm^−1^ marker peak which is only influenced by the electromagnetic field enhancement mechanism [29]. The red dotted line marks the Raman intensity level obtained for the non-treated Ag reference and the blue dotted lines mark the Raman intensity level of the water-treated substrate.

**Figure 4:**
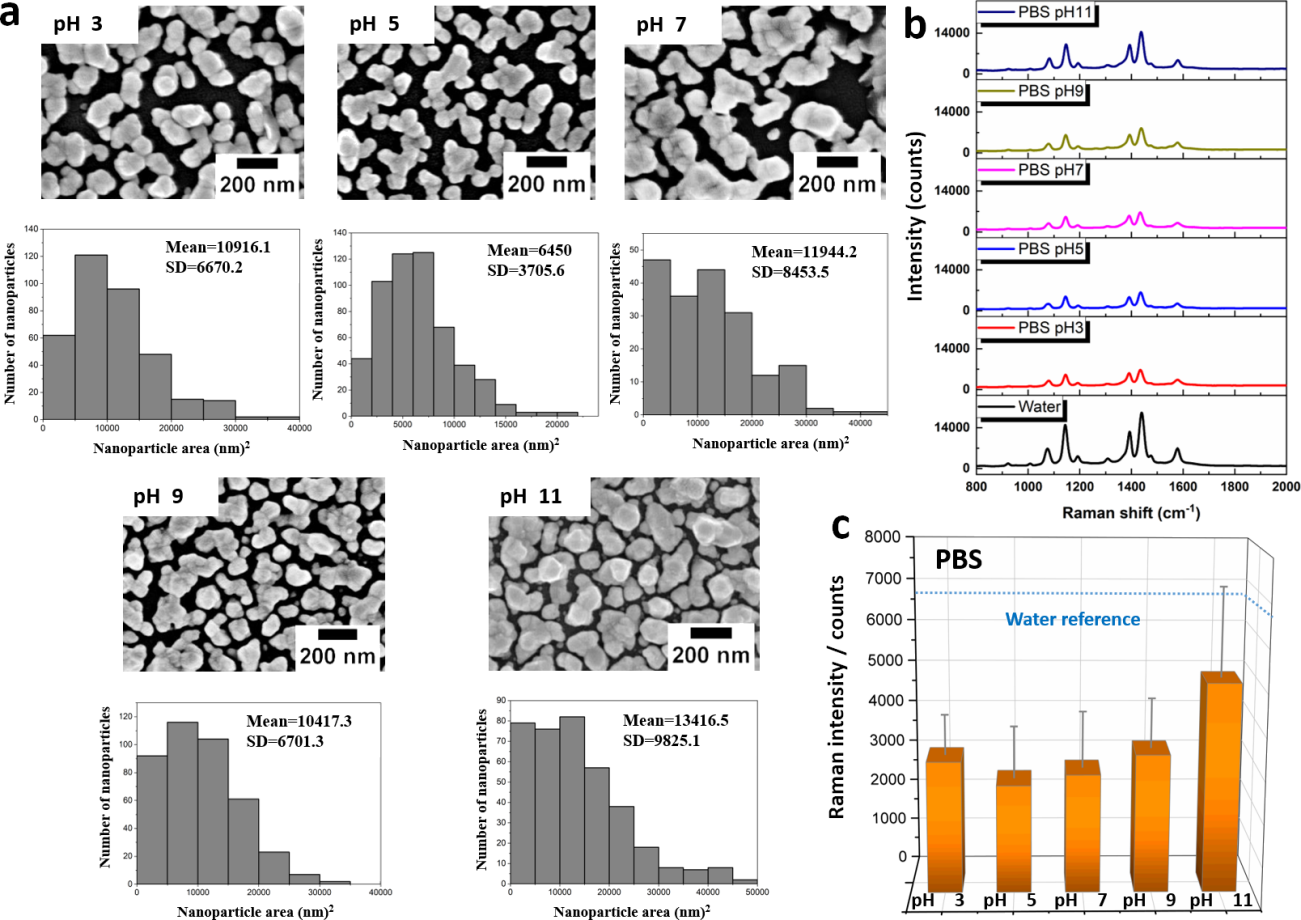
Stability test of microwave-fabricated SERS substrates in PBS solutions at different pH values. (a) SEM images of Ag nanoparticle coatings after 1 h of immersion in PBS and the corresponding particle size distributions. (b) SERS spectra of a monolayer of 4-ATP. The spectrum obtained after water treatment serves as a reference. (c) Error analysis of the 1078 cm^−1^ marker peak which is attributed to the electromagnetic field enhancement mechanism [29]. The blue dotted line marks the Raman intensity level obtained for 1 h of water treatment.

**Figure 5:**
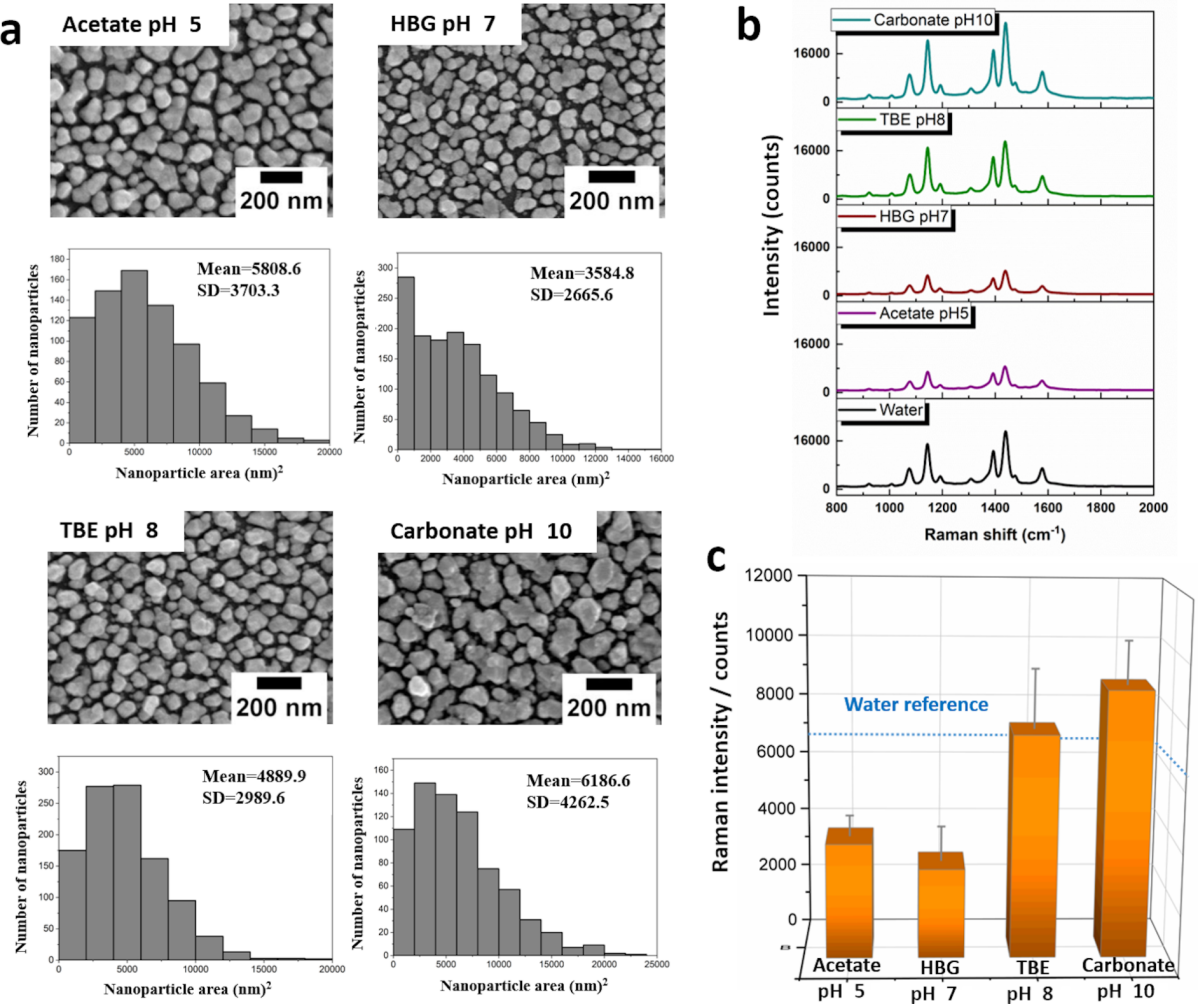
Stability test of microwave-fabricated SERS substrates for different buffer solutions with different pH values. (a) SEM images of Ag nanoparticle coatings after 1 h of immersion into different buffer solutions. (b) SERS spectra of a monolayer of 4-ATP. The spectrum obtained after water treatment serves as a reference. (c) Error analysis of the 1078 cm^−1^ marker peak which is attributed to the electromagnetic field enhancement mechanism [29]. The blue dotted line marks the Raman intensity level obtained for 1 h of water treatment.

The stability of the SERS substrates was tested in different solvents and biological buffer systems. The determination of the stability included two essential tests. First the substrates were immersed into the respective solvents or buffer solutions for 1 or 4 h, respectively. It is assumed that an incubation time of 1 h is sufficient for most measurements and reflects a time span during which typical SERS preparations/measurements are carried out. Thus, the main focus here was on the investigation of samples that have been immersed into different solutions for 1 h. In some cases, longer incubation times were also tested (these are summarized in Supporting Information File 1, Figure S3). After immersing the substrates into different solutions, all samples were thoroughly cleaned and dried in a stream of nitrogen to ensure equal treatment of all substrates. Afterward, the SERS substrates were split in half. One half of the substrates was subjected to morphological investigations of particle structure and density by SEM. The other half of the SERS substrate was coated with a monolayer of 4-ATP, which serves as a test monolayer to evaluate the Raman spectroscopic performance of the SERS substrates. After self-assembly of 4-ATP from an ethanolic solution, the substrates were carefully cleaned with ethanol to ensure a complete removal of molecules that were non-specifically attached to the surface. The formation of a monolayer is ensured by utilizing a thiol-functionalized test molecule to form a defined monomolecular dense layer of analyte on the surface. Additionally, 4-ATP only binds to silver nanoparticles and does not bind to the underlying glass substrate. The self-assembly time for 4-ATP solutions was kept short to avoid any influence of the 4-ATP solutions on the morphological changes of the nanoparticles. For this purpose, an incubation time to sufficiently form a monolayer on the silver nanoparticle-coated SERS substrates by Raman intensity measurements was chosen according to Figure S4 in Supporting Information File 1. The laser excitation was set to 532 nm which overlaps with the plasmon resonance of the SERS substrates [24]. After an incubation time of eight minutes, the intensity of the characteristic peak of 4-ATP located at 1442 cm^−1^ reached a maximum intensity which did not further increase after longer incubation times. From these results it was concluded that a complete monolayer of 4-ATP was formed on the silver nanoparticles. A reference spectrum of 4-ATP deposited on a freshly prepared, non-treated SERS substrate is depicted in Supporting Information File 1, Figure S5 and the corresponding peak assignment can be found in Supporting Information File 1, Table S1. The observed band structure clearly identifies bands at 1394, 1440, and 1580 cm^−1^ which are indicative of 4,4’-dimercaptoazobenzene (DMAB), which is formed by an oxidative transformation of 4-ATP on Ag nanoparticle surfaces at higher laser power densities [28]. The intensity of the band at 1078 cm^−1^ (C–S stretching mode) is specifically used here for the evaluation of the electromagnetic enhancement of the SERS substrates. This vibrational mode is considered to be insensitive to chemical enhancement [29] and only reflects the effect of the electromagnetic field enhancement, which we then relate to the morphological changes of our SERS substrates. The SEM images and the respective Raman investigations of the SERS substrates after treatment with different solutions are presented in Figures 3–5. Table 1 summarizes the determined key properties of the SERS substrates for all tested solvents and buffer systems to provide a comprehensive overview.

**Table 1:** Summary of the morphological properties and SERS activities of SERS substrates after treatment with different solvents. Raman intensities are reported relative to the respective reference substrates.

	Particle diameter equivalent (nm)	Standard deviation (+ nm)	Coverage (%) compared to ref.^a^	Raman intensity (%) compared to ref.^a^

as-prepared by MW (ref. for organic solvents)	67	54	81	100
methanol	75	61	81	64
ethanol	80	64	82	68
DMF	79	67	81	76
toluene	64	52	81	18
DMSO	82	65	82	121
water	87	69	82	58

water (ref. for buffer systems)	87	69	82	100
PBS (pH 3)	118	92	54	47
PBS (pH 5)	91	69	52	39
PBS (pH 7)	123	104	49	43
PBS (pH 9)	115	92	59	50
PBS (pH 11)	131	112	69	75
acetate (pH 5)	86	69	68	58
HBG (pH 7)	68	58	69	45
TBE (pH 8)	79	62	76	112
carbonate buffer (pH 10)	89	74	74	134

^a^Error bars are included in the respective histograms in Figures 3–5.

In a first set of experiments, the influence of five different organic solvents (methanol, ethanol, *N*,*N*-dimethylformamide (DMF), toluene, dimethyl sulfoxide (DMSO)) and water on the nanoparticle film morphology was investigated (Figure 3). The substrates were immersed into the different solvents for 1 and 4 h, respectively, and were subsequently analyzed by SEM and SERS. All corresponding sample morphologies found after an incubation time of 1 and 4 h are summarized in Supporting Information File 1, Figure S3. The morphology of the SERS substrates after an immersion time of 1 h does not significantly change as it can be observed from the corresponding SEM images as well as from the analysis of particle sizes and coverages, which all remain comparable to the as-prepared silver nanoparticle SERS substrates after their synthesis. While the particle size slightly increases in most cases, with an exception of the toluene treatment, the surface coverage remains identical for all samples. However, it is clearly observed that the obtained Raman intensities are significantly influenced by the immersion of the substrates into the respective solvents. Figure 3b and Figure 3c summarize the Raman activity of the SERS substrates after the treatment of the substrates with the individual organic solvents for 1 h and subsequent monolayer formation. All substrates still show a significant enhancement of the Raman signals; however, obvious deviations from the reference sample morphology are observed. While for methanol, ethanol, and DMF the reduction of the obtained Raman intensity decreases moderately, the Raman signals are strongly decreased by the toluene treatment. For the treatment with ethanol this might be related to the fact that the irregularly shaped Ag nanoparticles can be reshaped into more spherical nanoparticles after immersion into ethanol [30]. An evidence for this effect can be found in Figure S3 since after 4 h of immersion into ethanol more spherical nanoparticles were observed. The spherical nanoparticles apparently tend to generate fewer hot spots compared to irregularly shaped nanoparticles in the reference sample, which might result in a decrease in the Raman intensity. Similar reshaping was also observed in the SEM images of the samples treated with methanol and DMF (Supporting Information File 1, Figure S3) and more evidently in the sample treated with toluene, in which already after 1h a significant rounding of the particles was observed. However, it is unlikely that the significant decrease in the observed Raman intensities is solely explained by this effect and it is assumed that additional chemical effects play a role. It has been reported that the presence of surface-absorbed water can result in the deterioration of Ag nanoparticles which can seriously influence their stability [31]. In contrast, the treatment with DMSO resulted in a preservation or even increase in the Raman intensities. This might be explained by the fact that they retain their irregular nanoparticle structure (Supporting Information File 1, Figure S3). Moreover, DMSO can be considered as an active reducing agent for silver ions under suitable conditions [32]. Thus, Ag^+^ ions (which can be formed by the influence of water [31]) could be absorbed on the surface of the Ag nanoparticle, where they can be reduced by DMSO.

After immersion of the SERS substrates into water the decrease in the obtained Raman intensity is in the order of 43%, even though the particle sizes and coverage are similar to those of substrates immersed into other solvents, such as methanol, ethanol, and DMF. These initial studies already indicate the importance of the present study as a decrease in the SERS performance can evidently occur during sample preparation.

The use of SERS analysis is also increasing in bio analytics. As such, our studies were consequently expanded to investigate the influence of several buffer media commonly used in biological applications on the performance of the SERS substrates. These media frequently contain a complex mixture of different compounds and also have different pH values. The choice of a suitable media is of utmost importance for many biological investigations as it can alter the structure of biomolecules (e.g., proteins) [33]. We tested five commonly used buffer systems which include phosphate-buffered saline (PBS), HEPES-buffered glucose solution (HBG), tris-borate-EDTA (TBE), an acetate buffer, and a carbonate buffer. These buffer solutions feature different pH values.

PBS solutions are available in a variety of pH values and were chosen for an initial test to investigate the stability of SERS substrates at different pH values. For this study, and the subsequent investigation of other buffer systems, a water-treated SERS substrate (1 h of immersion time) was used as the reference since all investigated buffer solutions are water based (Figure 2b and Supporting Information File 1, Figure S6; for the following discussion all Raman intensity deviations are referenced to this sample). Figure 4a summarizes the key parameters of the morphology of the SERS substrates after immersion in the respective buffer solutions for 1 h. Figure 4b and Figure 4c depict the results of the corresponding SERS investigations. In contrast to the applied organic solvents used, PBS has a more significant influence on the morphology and coverage of the SERS substrates. In particular, under acidic conditions, a strong decrease in particle coverage and alterations of the shape of the particles are observed, which is reflected also by an increase in the obtained spherical analogue particle sizes (Table 1). The effect is more severe in acidic PBS solutions and under those conditions a strong decrease in the Raman enhancement compared to water is also observed. Changes in the morphology of the nanoparticle induced by immersion into basic PBS solutions is less pronounced. The highest Raman enhancement and least obvious changes in particle morphology are observed for the sample treated with PBS at pH 11. The highest Raman enhancement could be potentially associated here to smaller gap sizes, which favor the formation of hot spots to enhance the Raman intensities due to electromagnetic field enhancement. However, due to the rather strong decrease in the Raman intensities even on this sample, additional chemical changes can be assumed to be induced by the PBS solution itself.

In this context, it is reported that Ag nanoparticle suspensions can re-precipitate silver chloride on the surface of Ag nanoparticles when they are subjected to chloride-containing biological media [34]. The generation of AgCl leads, for example, to a partial passivation of the silver surface for further reactions. Energy-dispersive X-ray spectroscopy (EDX) was used to confirm the presence of chloride on the Ag SERS substrates treated with PBS at pH 5. These investigations provided evidence for the presence of Cl as shown in Supporting Information File 1, Figure S7. At the same time, the removal of Ag nanoparticles from the substrate, resulting in a lower particle coverage, may also be a result of the generation of AgCl as it would lead to a weaker interaction between the nanoparticles and the glass substrate. Moreover, it has been reported that H^+^ ions in buffer solutions play an important role in the oxidation of silver [35]. Therefore, SERS substrates treated with acidic or neutral PBS are more prone to oxidation and, as a consequence, can show lower Raman activities.

We expanded our study further by testing other relevant biological buffer solutions. The main ingredients of these buffer media are listed in Supporting Information File 1, Table S2.

Interestingly, it is observed that all other tested buffer systems (acetate buffer, HBG, TBE, and carbonate buffer; Figure 5b and Figure 5c) demostrated a better performance compared to the PBS systems in terms of their Raman enhancement. However, for acetate and HBG buffer treatment also significantly lower Raman intensities were observed with respect to the water-treated reference samples. In contrast, for TBE almost no change in the Raman activity was observed, and the Raman enhancement for the carbonate buffer system even increased above the level that was observed for the water-treated reference sample. At the same time, the alteration of the size of the spherical analogue particle sizes is less pronounced compared to the PBS buffer system and all samples only show a relatively small increase in particle size compared to the water-treated reference sample. Thus, the improved performance of the carbonate buffer cannot directly be assigned to the respective gap sizes. Since the carbonate buffer system provides a pH of 10, the previously observed beneficial effect of basic buffer systems can contribute to a better performance of the SERS substrates as the nanoparticles are apparently less prone to oxidation in buffer solutions with a high pH value [35].

However, additional effects may also contribute to the observed differences of the SERS performance in different buffer systems. These will critically depend on the compounds present in the buffer solutions and further in-depth investigations are required.

However, this initial study can serve as a guideline for the proper choice of preparation media for SERS substrates consisting of non-coated Ag nanoparticles. The presented investigations demonstrate the importance of considering these effects to ensure a good performance of SERS substrates, since already during the preparation step significant alterations of the electromagnetic field enhancement can be induced – either by changing the particle coverage or by inducing chemical changes of the silver nanoparticle substrates.

## Conclusion

We demonstrated the effect of different organic solvents, biological buffer systems, and water on the performance of silver nanoparticle-based SERS substrates and provide a guide for the choice of suitable preparation conditions for SERS investigations. The influence of different media on the performance of the SERS substrates could be rationalized by investigating the morphology and particle coverage of the SERS substrates after they have been immersed into those media. It was found that morphological changes and a significant decrease in the particle coverage is observed in particular for PBS systems, while organic solvents and other buffer systems only induce minor changes in the morphology and surface coverage of the nanoparticles. However, for some of these systems a decrease in the SERS performance is observed despite of the unchanged nanoparticle layer morphology. In these cases, chemical alterations of the surfaces of the nanoparticles are suspected to induce the alteration of the SERS enhancement of these substrates. DMSO and carbonate buffers at pH 10 showed the most promising results for maintaining the SERS activity of the substrates, while for other solvents a decrease in the Raman activity of the SERS substrates is frequently observed. A detailed investigation of the underlying processes is, in this respect, a logical step for further investigations.

## Experimental

### Chemicals

Silver acetate (≥99%), methanol, and DMSO were purchased from Sigma-Aldrich. 4-ATP and DMF were purchased from Alfa-Aesar. Toluene was purchased from Acros Organics and ethanol from VWR International GmbH. All the organic solvents (methanol, ethanol, DMF, toluene, and DMSO) were used without further purification. 4-ATP was also utilized as purchased.

### Preparation of surface-enhanced Raman spectroscopy substrates

A silver acetate precursor solution was used as a metal salt and ethanol was utilized as a reducing agent. Commercially available microscope glass coverslips, cut into rectangles with a dimension of 20 × 10 mm, were used as substrates. The glass substrates were first cleaned by washing with demineralized water and ethanol followed by plasma cleaning (argon plasma, 5 min, Diener Electronics). For all experiments, a single-mode microwave (Initiator, Biotage) was employed. In a typical batch synthesis of the SERS substrate, 12 mM of an aqueous solution of silver acetate was mixed with ethanol in a 2:1 ratio. Three substrates were placed in a 5 mL microwave vial which was filled up to 3 mL with water/ethanol/precursor solution. Subsequently, the microwave vial was capped with an aluminum crimp cap and a Teflon septum seal and was subjected to microwave irradiation. The microwave was set to operate at a constant irradiation power of 300 W. According to our previous investigations of the process parameters, the synthesis time was fixed to 2 min, which corresponds to the shortest time to yield a sufficient coverage with Ag nanoparticles with high homogeneity and SERS activity [24]. The chosen time of 2 min includes the heating ramp time and an additional cooling time of 2 min was required. Thus, the total microwave processing time was completed in 4 min. During a typical reaction, the temperature rises up to 140–160 °C and the pressure is between 7 to 9 bars. It is important to note that these temperature measurements provide values that are measured by an external IR sensor placed close to the reaction vial and do not represent the actual reaction temperature values inside the vial or on the substrate (i.e., near the Ag seeds or nanoparticles) during the processing time. Additionally, the filling level of the precursor reaction vial was kept below 3 mL to avoid excessive pressure buildup in the vial which could lead to an automatic shutdown of the microwave. The prepared substrates coated with a monolayer of Ag nanoparticles were immersed into various organic solvents (methanol, ethanol, DMF, toluene, and DMSO), buffer solutions (PBS solutions with pH values of 3, 5, 7, 9, and 11; pH 5 acetate buffer solution; pH 7 HBG buffer solutions; pH 8 TBE buffer solution, and pH 10 carbonate buffer solution) and deionized water (reference substrate) for 1 h. Afterward the substrates were carefully rinsed with water and dried in a stream of nitrogen to remove all residual solutions. The SERS substrates could also be subjected to ultrasonication to improve and support the cleaning process. Only excessive ultrasonication over longer periods of time leads to a sudden onset of nanoparticle detachment from the surfaces and must be avoided.

The substrates treated with different solutions were used for additional SEM investigation to determine the nanoparticle layers and their Raman activity. For this purpose, the substrates were split in half.

### Monolayer preparation

The 4-ATP monolayers were self-assembled onto cleaned and dried solvent and buffer-treated SERS substrates (see previous section) by immersing the prepared samples into 10^−4^ M 4-ATP/ethanol solutions for 8 min. Subsequently, the surfaces were cleaned with ethanol to remove the non-specifically attached 4-ATP molecules from the SERS substrates and were dried in a stream of N_2_.

### Raman measurements

SERS measurements were conducted at a laser excitation wavelength of 532 nm and all the experiments were carried out at a laser power of 0.2 mW and an integration time of 10 s on a Bruker Senterra confocal Raman microscope. For the evaluation of the SERS performance all investigations were performed with the same instrument and acquisition parameters. The positioning of the laser focus on the surface of the substrates was performed by adjusting the signal to its maximum intensity prior to the SERS measurements. This ensures comparable and defined focus conditions and enables the comparison of the SERS performance based on the peak intensities of the obtained SERS spectra. The error analysis at 1078 cm^−1^ for all SERS experiments was carried out on approximately 60 randomly chosen spots for each SERS sample.

### Scanning electron microscopy characterization

Before the SEM investigation, the SERS substrates were mounted onto sample holders with double-sided carbon tape. To improve the contrast and to reduce the potential charging effect, the samples were sputter-coated with platinum (Pt) with a coating thickness of 4 nm. The SEM imaging was performed with a field-emission scanning electron microscope (Sigma VP, Carl Zeiss AG, Jena, Germany) at an acceleration voltage of 5 kV utilizing the in-lens detector.

### Software and method

The automated image processing and analysis to determine the nanoparticle size distributions was performed by using the ImageJ 1.47v software. The workflow involved the application of a particle segmentation algorithm, which uses multiple watershed functions to derive the projected particle area. For this, the area occupied by the nanoparticles is identified by doing a manual threshold, which creates a binary image. The particles on the edges of the images were excluded for data extraction. Finally, a histogram of the area-equivalent diameter (AED) *d*_AED_ = 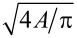 was plotted.

## Supporting Information

The supporting information includes the following materials: a diagram monitoring the essential microwave parameters during synthesis (Figure S1), a comparison of the substrates derived from two different batches (Figure S2), a summary of the SERS substrate morphologies after an immersion time of 1 and 4 h for the treatment with organic solvents and PBS buffers (Figure S3), the calibration curve to obtain a monolayer of 4-ATP (Figure S4), a reference Raman spectrum of 4-ATP self-assembled on a non-treated substrate (Figure S5), a table indicating the corresponding peak assignment (Table S1), a reference Raman spectrum of 4-ATP acquired on a water-treated substrate (Figure S6), EDX spectra obtained after PBS treatment at pH 5 (Figure S7 and a list summarizing the typical components of the utilized buffer systems (Table S2).

File 1 Additional figures and tables.
