# Supplementary material for: On the stability of microwave-fabricated SERS substrates – chemical and morphological considerations
Source: Beilstein J Nanotechnol. 2021 Jun 11;12:541–51. doi: 10.3762/bjnano.12.44 (PMC8204127; doi:10.3762/bjnano.12.44)
Supplement: File 1 — Additional figures and tables. [file Beilstein_J_Nanotechnol-12-541-s001.pdf]

BEILSTEIN JOURNAL OF NANOTECHNOLOGY

Supporting Information

for

On the stability of microwave-fabricated SERS substrates – chemical and morphological considerations

Limin Wang, Aisha Adebola Womiloju, Christiane Höppener, Ulrich S. Schubert and Stephanie Hoeppener

Beilstein J. Nanotechnol. **2021,** *12,* 541–551. doi:10.3762/bjnano.12.44

Additional figures and tables

License and Terms: This is a supporting information file under the terms of the Creative Commons Attribution License (https://creativecommons.org/licenses/by/4.0). Please note that the reuse, redistribution and reproduction in particular requires that the author(s) and source are credited and that individual graphics may be subject to special legal provisions.
The license is subject to the *Beilstein Journal of Nanotechnology* terms and conditions: (https://www.beilstein-journals.org/bjnano/terms)

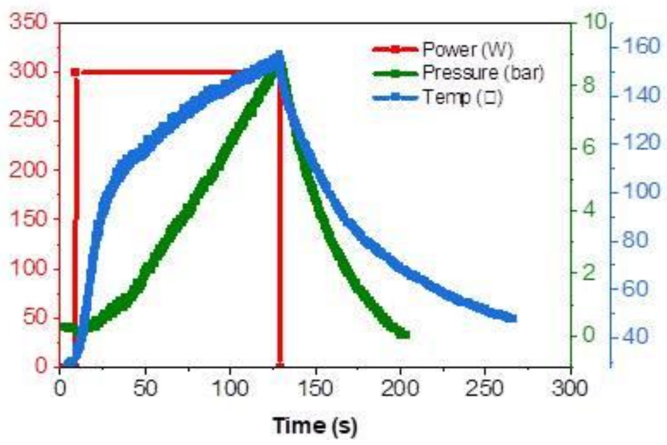

Figure S1: Representative diagram depicting the power, pressure, and temperature measured during the microwave-assisted coating of the glass substrate.

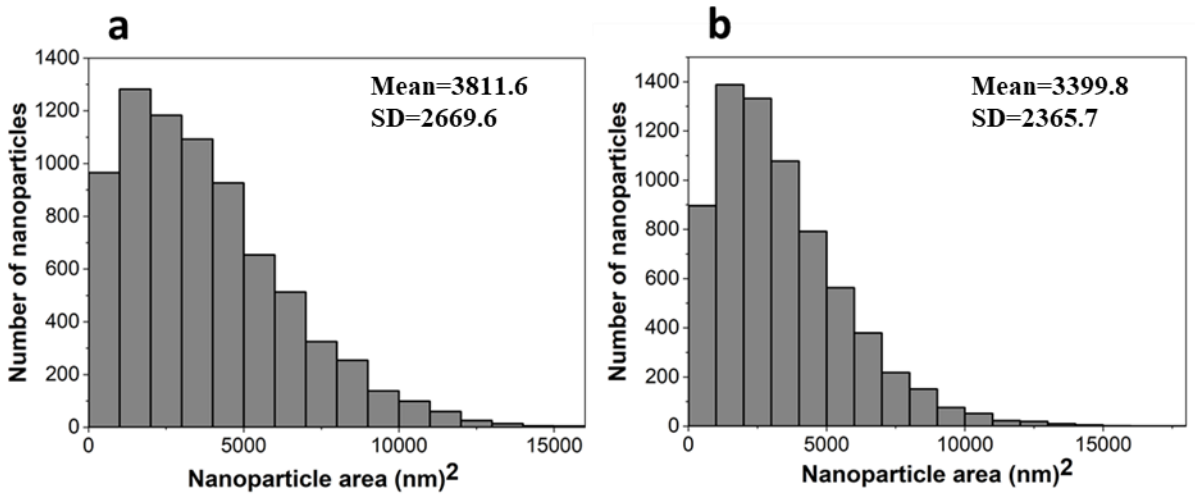

Figure S2: Histograms of SERS substrates from different batches after their preparation: (a) batch 1 and (b) batch 2.

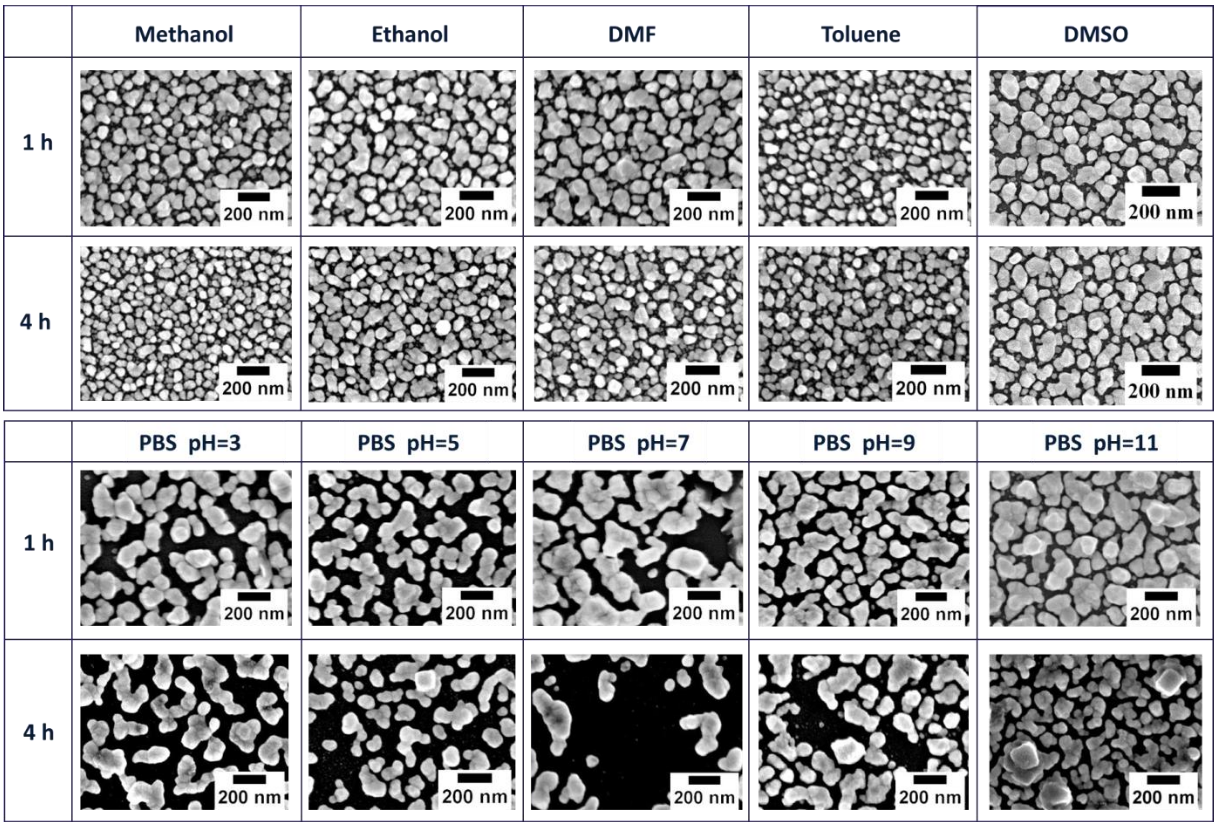

Figure S3: SEM images of the SERS substrates immersed into different organic solvents and PBS solutions for 1 and 4 h, respectively.

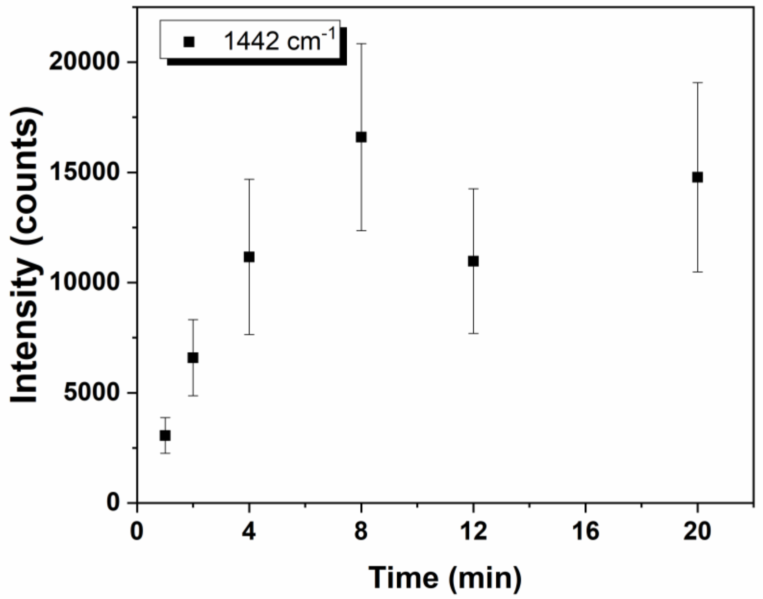

Figure S4: Raman intensities found for the marker peak at 1442 cm^{-1} of a monolayer prepared from 10^{-4} M 4-ATP. After eight minutes the peak intensity reaches a plateau, indicating the formation of a complete monolayer on the SERS substrates.

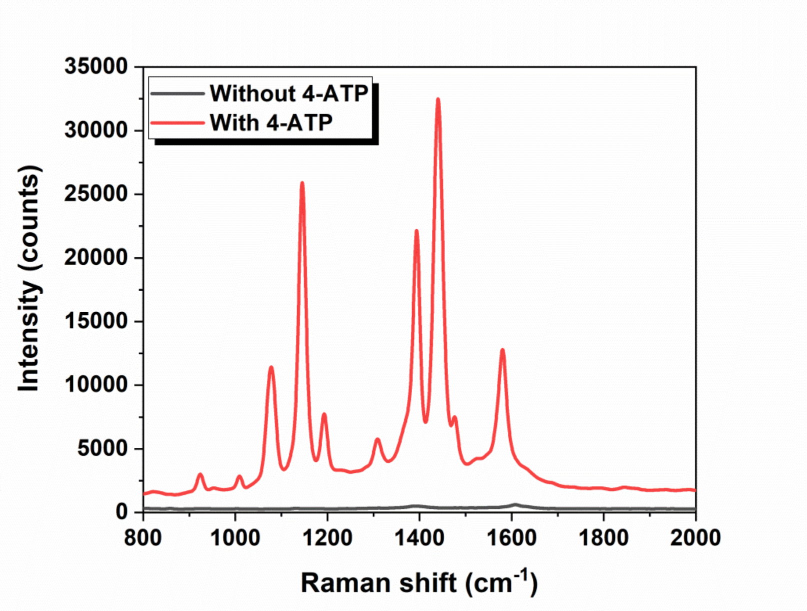

Figure S5: Reference Raman spectra of a 4-ATP-coated, non-treated, clean SERS substrate (red) and the Raman spectrum of the SERS substrate only (black). The corresponding peak assignment is provided in Table S1.

Table S1: Peak assignment for the SERS spectrum shown in Figure S5 [1,2]. A dimerization process takes place and leads to the transformation of 4-ATP into 4,4'-dimercaptoazobenzene (DMAB) as seen in the appearance of the b_2 modes or a_g, respectively [1].

**SERS band frequency/cm^{-1}**	**Assignment (4-APT/DMAB)**
**1580**	ν(CC), b_2/a_g mode
**1440**	δ(CH) + ν(CC), b_2 /a_g mode
**1394**	ν(CC) + δ(CH), b_2 /a_g mode
**1145**	δ(CH), a_1 mode
**1078**	ν(CS), a_1 mode

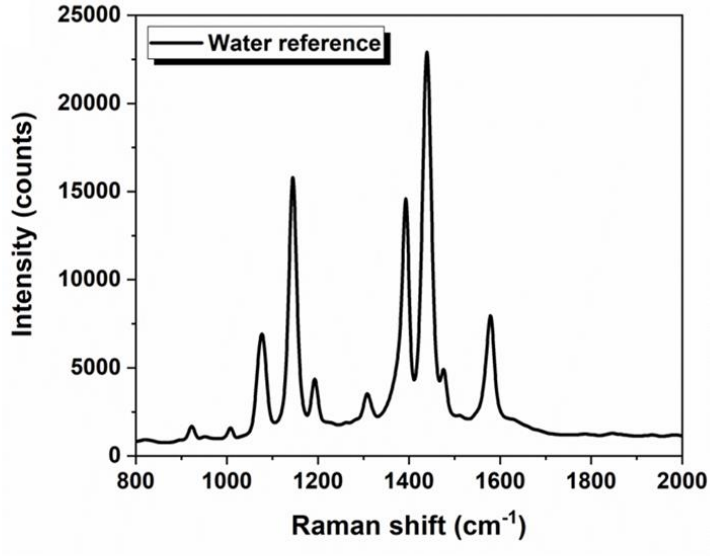

Figure S6: Reference Raman spectrum of a monolayer of 4-ATP self-assembled on a water-treated SERS substrate.

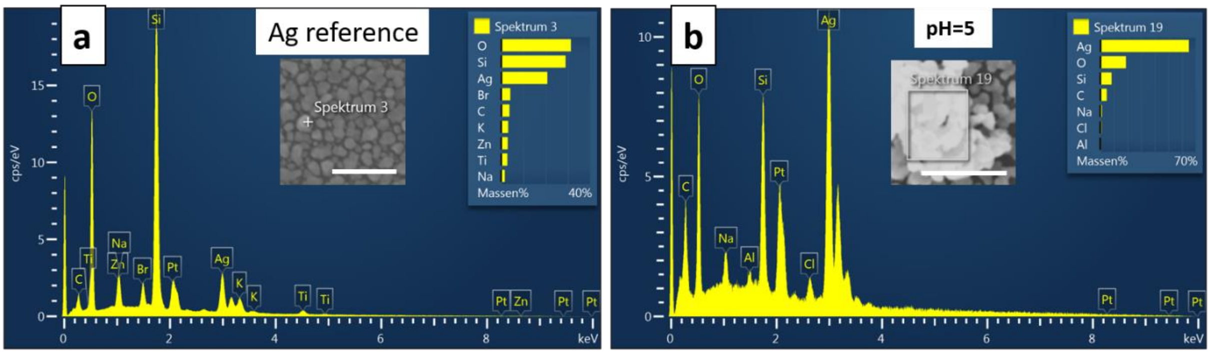

Figure S7: EDX elemental analysis of the SERS substrate without treatment (a) and treated with PBS buffer of pH 5 (b). The insets represent the SEM images. Scale bar: 500 nm. The elements O, Si, C, Na, and Al in (b) could be attributed to the glass substrate.

Table S2: Main components of different buffer solutions.

**Different buffer solutions**	**Ingredients**	**Cations**	**Anions**
**PBS pH 3**	NaCl, KCl, NaH_2PO_4, KH_2PO_4	Na^+, K^+	Cl^-, $H_2PO_4^-$
**PBS pH 5**	NaCl, KCl, NaH_2PO_4, KH_2PO_4	Na^+, K^+	Cl^-, $H_2PO_4^-$
**Acetate pH 5**	CH_3COONa, CH_3COOH	Na^+	CH_3COO^-
**PBS pH 7**	NaCl, KCl, Na_2HPO_4, KH_2PO_4	Na^+, K^+	Cl^-, HPO_4^{2-}, $H_2PO_4^-$
**HBG pH 7**	HEPES, glucose		
**TBE pH 8**	Tris base, boric acid, EDTA		
**PBS pH 9**	NaCl, KCl, Na_2HPO_4, K_2HPO_4	Na^+, K^+	Cl^-, HPO_4^{2-}
**Carbonate pH 10**	$NaHCO_3$, Na_2CO_3	Na^+	HCO_3^-, CO_3^{2-}
**PBS pH 11**	NaCl, KCl, Na_2HPO_4, K_2HPO_4	Na^+, K^+	Cl^-, HPO_4^{2-}

[1] Huang, Y.-F.; Wu, D.-Y.; Zhu, H.-P.; Zhao, L.-B.; Liu, G.-K.; Ren, B.; Tian, Z.-Q. *Phys. Chem. Chem.l Phys.,* **2012,** *14*, 8485–8497.

[2] Hu, X.; Wang, T.; Wang, L.; Dong, S. *J. Phys. Chem. C* **2007,** *111*, 6962–6969.
